# Supplementary material for: Diflunisal and Analogue Pharmacophores Mediating Suppression of Virulence Phenotypes in Staphylococcus aureus
Source: Antibiotics (Basel). 2023 Jul 12;12(7):1180. doi: 10.3390/antibiotics12071180 (PMC10376238; doi:10.3390/antibiotics12071180)
Supplement: Supplementary file 1 [file antibiotics-12-01180-s001.zip › antibiotics-2435590-supplementary.pdf]

Chan et al. Supplemental Figure S1

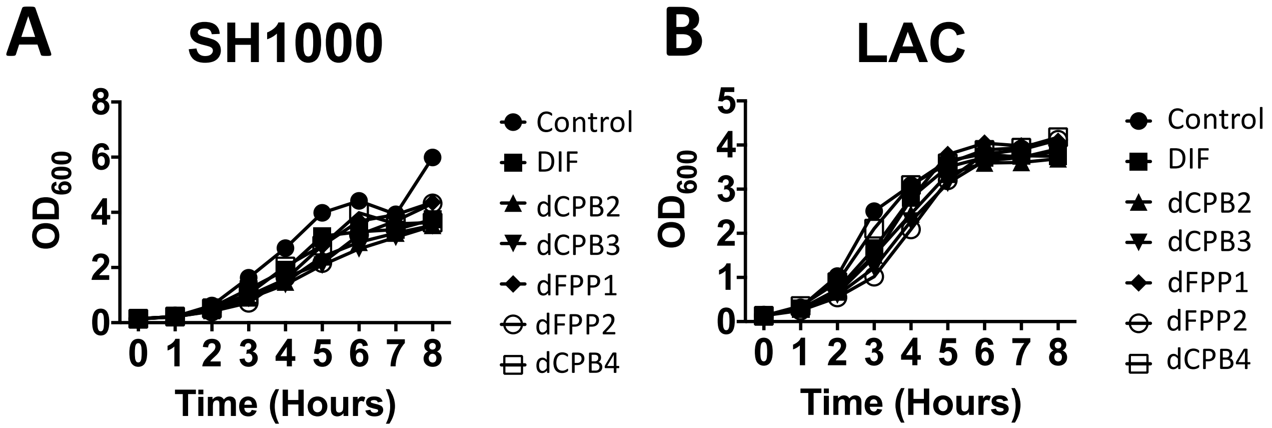

Supplemental Figure S1. DIF and structural analogues do not impact growth in *S. aureus* strains. Strains were grown in tryptic soy broth with or without DIF or analogues. Cultures were measure every hour to measure growth.

Chan et al. Supplemental Figure S2

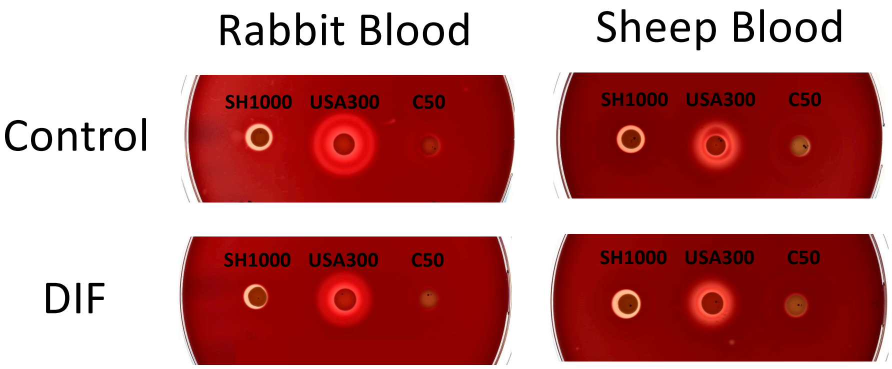

Supplemental Figure S2. Hemolysis assays performed on rabbit vs. sheep blood agar showed similar reductions when treated with DIF.

Chan et al. Supplemental Table S1

S. aureus Strain

		MSSA	COL	LAC	MW2	Mu3	Mu50	C15	C16
Hydroxy Phenyl BZA	OHPB1	0.975	0.529	0.691	0.109	0.688	0.862	0.084	0.481
	OHPB2	0.079	0.427	0.177	0.059	0.375	0.598	0.018	0.420
Fluorophenyl BA	FPB1	0.252	0.627	0.223	0.137	0.067	0.240	0.047	0.051
Difluorophenyl BZA	dFPB1	0.119	0.037	0.963	0.679	0.082	0.295	0.658	0.039
	dFPB2	0.085	0.447	0.867	0.162	0.156	0.289	0.459	0.451
	dFPB3	0.013	0.129	0.013	0.088	0.008	0.021	0.011	0.001
	dFPB4	0.225	0.166	0.343	0.236	0.520	0.912	0.070	0.585
	dFPB5	0.128	0.384	0.071	0.034	0.020	0.050	0.011	0.021
	dFPB6	0.008	0.442	0.013	0.003	0.016	0.025	0.014	0.069
Difluorophenyl Phenol	dFPP1	0.685	0.689	0.021	0.765	0.173	0.361	0.188	0.731
	dFPP2	0.084	0.308	0.073	0.370	0.884	0.537	0.170	0.654
Dichlorophenyl BZA	dCPB1	0.213	0.189	0.074	0.273	0.163	0.052	0.028	0.050
	dCPB2	0.089	0.050	0.006	0.059	0.015	0.028	0.008	0.003
	dCPB3	0.465	0.089	0.452	0.459	0.378	0.463	0.175	0.934
	dCPB4	0.255	0.142	0.012	0.612	0.198	0.049	0.021	0.001
	dCPB5	0.481	0.607	0.995	0.104	0.790	0.926	0.393	0.393
	dCPB6	0.013	0.070	0.873	0.091	0.997	0.826	0.553	0.996
	dCPB7	0.004	0.710	0.238	0.093	0.840	0.653	0.746	0.733
BZA	BZA	0.005	0.404	0.290	0.038	0.102	0.372	0.041	0.188
Dichloro BZA	dCB1	0.018	0.459	0.275	0.067	0.451	0.897	0.124	0.426
	dCB2	0.071	0.367	0.323	0.042	0.734	0.681	0.038	0.412
	dCB3	0.004	0.260	0.352	0.004	0.544	0.469	0.006	0.346
Difluoro BZA	dFB1	0.038	0.427	0.264	0.068	0.541	0.406	0.051	0.221
	dFB2	0.009	0.353	0.084	0.055	0.210	0.848	0.059	0.141
	dFB3	0.032	0.394	0.092	0.040	0.235	0.708	0.043	0.232
Chlorofluoro BZA	CFB	0.027	0.405	0.085	0.057	0.172	0.246	0.066	0.068

Hemolysis P-values: DIF vs. Analogues

Supplemental Table S1. Student's t-test P-values reveal compounds that perform significantly better than DIF. P-values of DIF vs. structural analogues in hemolysis (A), proteolysis (B) and biofilm (C) assays were calculated for significantly different impacts. Values highlighted in green indicate significantly decreased ($P < 0.05$) virulence phenotypes as compared to DIF, whereas values highlighted in red indicate significantly increased ($P < 0.05$) virulence phenotypes.

Chan et al. Supplemental Table S2

S. aureus Strain

		MSSA	COL	LAC	MW2	Mu3	Mu50	C15	C16
Hydroxy Phenyl BZA	OHPB1	1.000	1.000	0.316	1.000	1.000	0.184	0.423	0.423
	OHPB2	1.000	1.000	1.000	1.000	1.000	0.349	1.000	1.000
Fluorophenyl BA	FPB1	0.270	0.000	0.005	0.038	0.085	0.028	0.009	0.001
Difluorophenyl BZA	dFPB1	0.363	0.001	0.003	0.001	1.000	0.276	0.000	0.001
	dFPB2	0.159	0.029	0.006	0.014	0.003	0.049	0.001	0.002
	dFPB3	0.058	0.003	0.000	0.000	0.000	0.009	0.000	0.000
	dFPB4	1.000	0.001	0.000	0.000	0.138	0.127	0.068	0.008
	dFPB5	0.363	0.004	0.000	0.001	0.046	0.090	0.017	0.076
	dFPB6	0.321	0.003	0.000	0.003	0.007	0.042	0.002	0.001
Difluorophenyl Phenol	dFPP1	0.229	0.001	0.000	0.003	0.002	0.030	0.001	0.004
	dFPP2	0.271	0.002	0.000	0.001	0.001	0.013	0.001	0.008
Dichlorophenyl BZA	dCPB1	0.363	0.005	0.001	0.106	1.000	0.079	0.001	0.115
	dCPB2	1.000	0.363	0.219	1.000	1.000	1.000	0.258	0.363
	dCPB3	1.000	1.000	0.180	0.363	1.000	1.000	1.000	1.000
	dCPB4	1.000	0.374	0.374	1.000	1.000	0.374	0.374	1.000
	dCPB5	1.000	0.090	0.005	0.022	1.000	1.000	0.092	0.096
	dCPB6	1.000	0.214	0.026	0.104	1.000	0.222	0.252	0.423
	dCPB7	1.000	0.004	0.036	0.031	1.000	1.000	0.185	0.188
BZA	B	1.000	0.029	0.007	0.008	0.110	0.022	0.073	0.090
Dichloro BZA	dCB1	1.000	0.050	0.018	0.023	0.211	0.045	0.067	0.091
	dCB2	1.000	0.006	0.009	0.041	0.233	0.030	0.066	0.092
	dCB3	1.000	0.050	0.005	0.012	0.117	0.009	0.068	0.156
Difluoro BZA	dFB1	1.000	0.056	0.005	0.006	0.142	0.059	0.122	0.143
	dFB2	1.000	0.034	0.002	0.011	0.169	0.002	0.054	0.077
	dFB3	1.000	0.032	0.004	0.005	0.110	0.003	0.018	0.040
Chlorofluoro BZA	CFB	1.000	0.012	0.014	0.015	0.158	0.008	0.045	0.073

Proteolysis P-values: DIF vs. Analogues

Supplemental Table S2. Student's t-test P-values reveal compounds that perform significantly better than DIF. P-values of DIF vs. structural analogues in hemolysis (A), proteolysis (B) and biofilm (C) assays were calculated for significantly different impacts. Values highlighted in green indicate significantly decreased ($P < 0.05$) virulence phenotypes as compared to DIF, whereas values highlighted in red indicate significantly increased ($P < 0.05$) virulence phenotypes.

Chan et al. Supplemental Table S3

S. aureus Strain

		MSSA	COL	LAC	MW2	Mu3	Mu50	C15	C16
Hydroxy Phenyl BZA	OHPB1	0.127	0.022	0.051	0.001	0.649	0.419	0.402	0.022
	OHPB2	0.014	0.017	0.081	0.005	0.317	0.136	0.160	0.055
Fluorophenyl BA	FPB1	0.257	0.053	0.010	0.025	0.011	0.015	0.064	0.111
Difluorophenyl BZA	dFPB1	0.110	0.043	0.042	0.019	0.081	0.045	0.016	0.050
	dFPB2	0.114	0.011	0.001	0.077	0.039	0.018	0.137	0.124
	dFPB3	0.053	0.016	0.098	0.061	0.078	0.003	0.011	0.083
	dFPB4	0.222	0.043	0.018	0.232	0.069	0.047	0.099	0.085
	dFPB5	0.100	0.001	0.148	0.099	0.012	0.021	0.032	0.028
	dFPB6	0.101	0.030	0.082	0.011	0.052	0.007	0.127	0.199
Difluorophenyl Phenol	dFPP1	0.121	0.009	0.128	0.059	0.291	0.018	0.518	0.674
	dFPP2	0.032	0.012	0.180	0.019	0.112	0.094	0.101	0.018
Dichlorophenyl BZA	dCPB1	0.092	0.053	0.093	0.356	0.479	0.000	0.575	0.293
	dCPB2	0.032	0.133	0.005	0.024	0.218	0.705	0.471	0.005
	dCPB3	0.392	0.069	0.696	0.053	0.552	0.003	0.092	0.112
	dCPB4	0.130	0.150	0.140	0.888	0.062	0.051	0.439	0.100
	dCPB5	0.002	0.014	0.035	0.003	0.482	0.655	0.922	0.045
	dCPB6	0.012	0.056	0.112	0.000	0.435	0.611	0.652	0.025
	dCPB7	0.078	0.002	0.085	0.002	0.955	0.270	0.307	0.203
BZA	BZA	0.037	0.037	0.045	0.001	0.467	0.026	0.220	0.062
Dichloro BZA	dCB1	0.065	0.022	0.074	0.007	0.430	0.045	0.164	0.027
	dCB2	0.032	0.017	0.132	0.011	0.613	0.236	0.448	0.033
	dCB3	0.074	0.007	0.040	0.005	0.318	0.145	0.146	0.034
Difluoro BZA	dFB1	0.063	0.005	0.049	0.007	0.304	0.083	0.312	0.013
	dFB2	0.065	0.029	0.081	0.004	0.350	0.017	0.190	0.051
	dFB3	0.279	0.013	0.050	0.001	0.306	0.060	0.176	0.004
Chlorofluoro BZA	CFB	0.064	0.016	0.056	0.004	0.168	0.088	0.183	0.010

Biofilm P-values: DIF vs. Analogues

Supplemental Table S3. Student's t-test P-values reveal compounds that perform significantly better than DIF. P-values of DIF vs. structural analogues in hemolysis (A), proteolysis (B) and biofilm (C) assays were calculated for significantly different impacts. Values highlighted in green indicate significantly decreased ($P < 0.05$) virulence phenotypes as compared to DIF, whereas values highlighted in red indicate significantly increased ($P < 0.05$) virulence phenotypes.

Chan et al. Supplemental Table S4A – DIF and structural analogues differentially mitigate virulence in *S. aureus*

Compound Name	*Structure & M.W.*	% Hemolysis (H) / Proteolysis (P) / Biofilm (B) vs. Control (SD)								
			**MSSA**	**COL**	**LAC**	**MW2**	**Mu3**	**Mu50**	**C15**	**C16**
Diflunisal 2-hydroxy-5-(2,4-difluorophenyl) benzoic acid	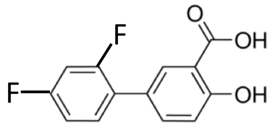 *250.2*	H	41.6 [27.8]	66.9 [35.8]	86.2 [18.7]	65.7 [25.0]	87.6 [23.3]	81.0 [36.7]	73.4 [18.7]	81.9 [27.8]
		P	0.0 [0]	0.0 [0]	0.0 [0]	0.0 [0]	0.0 [0]	0.0 [0]	0.0 [0]	0.0 [0]
		B	64.4 [20.4]	46.3 [2.9]	17.3 [9.5]	8.9 [2.6]	63.0 [33.8]	72.9 [7.1]	65.1 [3.4]	45.1 [14.7]
OHPB1 2-Hydroxy-4-phenylbenzoic acid	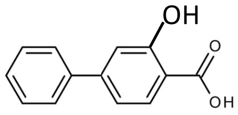 *214.2*	H	37.7 [56.5]	89.6 [0.2]	89.5 [19.0]	170.9 [66.5]	89.0 [43.4]	84.2 [27.1]	93.2 [15.7]	100.0 [28.3]
		P	0.0 [0]	0.0 [0]	15.5 [20.3]	0.0 [0]	0.0 [0]	3.3 [2.9]	12.7 [22.0]	8.1 [14.1]
		B	79.8 [11.9]	**84.9 [10.7]**	99.4 [47.6]	**81.2 [17.3]**	85.3 [20.8]	73.7 [23.0]	73.7 [27.0]	**99.1 [14.9]**
OHPB2 2-Hydroxy-5-phenylbenzoic acid	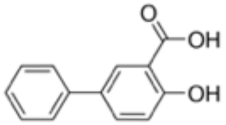 *214.2*	H	135.4 [26.3]	103.2 [0.5]	102.1 [13.9]	203.8 [71.4]	107.0 [43.7]	91.4 [20.4]	**95.5 [16.0]**	96.5 [20.5]
		P	0.0 [0]	0.0 [0]	0.0 [0]	0.0 [0]	0.0 [0]	3.0 [4.3]	0.0 [0]	0.0 [0]
		B	**293.2 [59.0]**	**106.9 [10.1]**	75.8 [31.7]	**86.6 [15.5]**	97.6 [14.4]	92.0 [15.9]	91.1 [15.9]	106.4 [27.4]
FPB1 4-(2-fluorophenyl) benzoic acid	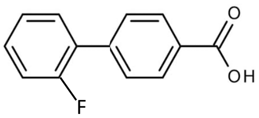 *216.2*	H	56.5 [32.3]	76.8 [41.0]	106.9 [26.5]	127.2 [92.1]	114.1 [33.7]	101.2 [44.6]	**110.4 [24.4]**	110.5 [29.3]
		P	316.9 [627]	**230.0 [48.5]**	**148.5 [77.2]**	**208.0 [182]**	41.3 [47.3]	**126.0 [101]**	**144.3 [85.6]**	**104.7 [40.2]**
		B	85.9 [30.8]	54.6 [6.3]	**27.8 [11.3]**	**19.0 [2.8]**	**102.1 [40.6]**	**92.3 [6.0]**	77.9 [4.2]	76.4 [18.0]
dFPB1 3-(2,4-difluorophenyl) benzoic acid	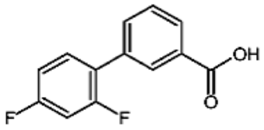 *234.2*	H	55.9 [27.7]	**53.8 [32.0]**	85.9 [16.4]	63.2 [24.1]	79.7 [24.8]	71.3 [37.7]	77.9 [15.9]	**71.8 [27.5]**
		P	37.4 [91.5]	**147.5 [48.5]**	**109.0 [49.3]**	**126.0 [42.3]**	0.0 [0]	116.7 [234]	**119.1 [27.1]**	**121.3 [38.6]**
		B	78.0 [18.6]	**53.3 [2.9]**	**24.2 [11.1]**	**25.9 [6.6]**	101.9 [53.5]	**103.1 [7.8]**	**100.9 [10.8]**	95.7 [28.2]
dFPB2 4-(2,4-difluorophenyl) benzoic acid	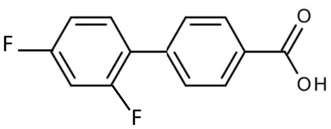 *234.2*	H	74.4 [46.5]	50.3 [45.5]	85.0 [14.6]	79.9 [21.9]	64.4 [26.3]	67.2 [38.4]	83.3 [29.7]	74.8 [30.7]
		P	159.5 [236]	**142.9 [115]**	**131.4 [71.2]**	**122.2 [81.2]**	**58.5 [27.6]**	**186.9 [178]**	**117.3 [41.6]**	**121.9 [51.4]**
		B	74.8 [20.3]	**72.6 [7.6]**	**48.1 [8.3]**	26.2 [11.2]	**106.1 [45.1]**	**98.0 [6.9]**	101.6 [24.0]	84.2 [25.9]

Supplemental Table S4. Hemolysis (H), proteolysis (P) and biofilm (B) for DIF and structural analogues are represented as percent of control. Mean values and standard deviation (SD) for DIF and each analogues are shown for DIF and each structural analogue. Values of structural analogues that significantly ($P < 0.05$; student's t-test) differed from DIF are indicated in bold font.

Chan et al. Supplemental Table S4B – DIF and structural analogues differentially mitigate virulence in *S. aureus*

Compound Name	*Structure & M.W.*	% Hemolysis (H) / Proteolysis (P) vs. Control (St. Dev.)								
			MSSA	COL	LAC	MW2	Mu3	Mu50	C15	C16
dFPB3<br>2-(2,4-difluorophenyl) benzoic acid	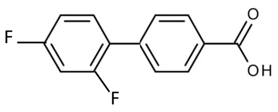<br>*234.2*	H	**77.2 [27.5]**	108.9 [37.7]	**105.1 [15.4]**	165.6 [142]	**121.0 [39.1]**	**122.1 [68.7]**	**105.2 [14.7]**	**112.1 [35.0]**
		P	137.0 [137]	**114.8 [51.2]**	**97.8 [19.0]**	**70.2 [17.6]**	**84.3 [22.1]**	**193.8 [115]**	**120.0 [26.2]**	**123.8 [37.8]**
		B	89.1 [23.8]	**83.4 [10.2]**	91.2 [52.1]	101.6 [43.7]	145.5 [76.2]	**120.7 [11.7]**	**142.8 [12.2]**	102.3 [32.0]
dFPB4<br>4-(3,5-difluorophenyl) benzoic acid	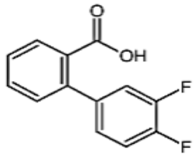<br>*234.2*	H	60.7 [37.1]	86.8 [40.4]	94.5 [12.1]	60.9 [22.7]	82.6 [35.5]	79.8 [50.7]	92.6 [25.1]	87.8 [44.8]
		P	0.0 [0.0]	**257.5 [92.9]**	**131.0 [31.5]**	**147.4 [42.6]**	18.9 [26.2]	126.9 [170]	177.3 [187]	**83.7 [48.6]**
		B	86.3 [38.4]	**59.3 [2.0]**	**28.5 [10.4]**	14.6 [7.9]	89.3 [43.7]	**87.0 [7.6]**	89.4 [16.5]	75.2 [17.3]
dFPB5<br>4-(2,3-difluorophenyl) benzoic acid	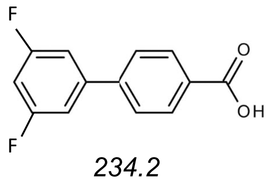<br>*234.2*	H	66.1 [40.2]	82.2 [44.5]	109.6 [17.2]	**88.2 [36]**	**126.5 [34.9]**	106.5 [49.5]	**104.5 [5.5]**	**104.1 [33.9]**
		P	120.1 [294]	**236.7 [115]**	**132.4 [27.8]**	**172.9 [56.5]**	**41.8 [38.7]**	157.9 [184]	**157.8 [109]**	199.4 [219]
		B	90.4 [10.7]	**64.7 [3.3]**	37.8 [19.2]	19.6 [8.8]	**132.5 [41.8]**	**101.5 [2.3]**	**108.1 [16.2]**	**89.1 [17.1]**
dFPB6<br>4-(2,6-difluorophenyl) benzoic acid	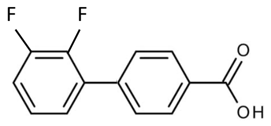<br>*234.2*	H	**102.8 [40.7]**	79.6 [38.6]	**126.0 [19.4]**	**145.9 [52.8]**	**142.1 [49.6]**	**118.0 [46.7]**	**114.3 [19.4]**	127.7 [59.7]
		P	417.1 [929]	**213.5 [96.6]**	**134.3 [31.0]**	**185.1 [86.4]**	**72.0 [40.2]**	**223.9 [202]**	**134.0 [57.7]**	**107.2 [39.6]**
		B	92.1 [32.6]	**76.6 [6.8]**	75.2 [40.2]	**34.7 [3.3]**	128.3 [60.5]	**100.8 [9.4]**	78.2 [5.7]	62.6 [11.1]
dFPP1<br>3-(2,4-difluorophenyl) phenol	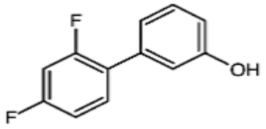<br>*206.2*	H	48.0 [46.3]	57.3 [48.9]	**110.5 [23.3]**	70.2 [48.9]	105.8 [45.3]	104.1 [62.6]	90.3 [27.0]	89.1 [50.0]
		P	93.2 [166]	**125.5 [48.3]**	**126.8 [23.7]**	**78.7 [34.6]**	**71.6 [28.2]**	**205.3 [167]**	**100.3 [35.5]**	**79.7 [39.3]**
		B	48.8 [14.4]	**65.0 [3.0]**	45.7 [28.4]	11.5 [2.5]	58.8 [31.1]	**87.5 [5.4]**	70.6 [10.0]	43.8 [13.0]
dFPP2<br>3-(2,3-difluorophenyl) phenol	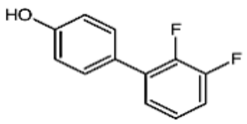<br>*206.2*	H	64.2 [29.2]	45.6 [37.1]	102.0 [15.2]	76.1 [37.7]	90.0 [48.9]	90.5 [57.1]	88.6 [19.9]	77.2 [42.8]
		P	101.4 [201]	**124.6 [51.3]**	**109.2 [24.3]**	**65.5 [22.0]**	**74.1 [23.2]**	**169.7 [111]**	**92.1 [28.3]**	**74.2 [42.6]**
		B	**8.3 [3.0]**	**102.3 [13.5]**	26.3 [17.2]	**18.2 [4.4]**	108.4 [61.7]	150.4 [51.3]	53.4 [9.2]	**51.3 [13.8]**

Supplemental Table S4. Hemolysis (H), proteolysis (P) and biofilm (B) for DIF and structural analogues are represented as percent of control. Mean values and standard deviation (SD) for DIF and each analogues are shown for DIF and each structural analogue. Values of structural analogues that significantly ($P < 0.05$; student's t-test) differed from DIF are indicated in bold font.

Chan et al. Supplemental Table S4C – DIF and structural analogues differentially mitigate virulence in *S. aureus*

Compound Name	*Structure & M.W.*	% Hemolysis (H) / Proteolysis (P) vs. Control (St. Dev.)								
			MSSA	COL	LAC	MW2	Mu3	Mu50	C15	C16
dCPB1 2-(3,5-dichlorophenyl) benzoic acid	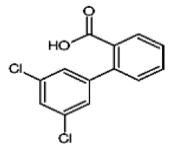 *267.1*	H	59.7 [41.6]	94.4 [42.4]	107.2 [29.2]	81.4 [37.9]	108.9 [56.8]	117.2 [72.1]	**108.6 [28.4]**	112.0 [51.8]
		P	305.6 [749]	**59.7 [30.2]**	**72.2 [26.5]**	22.1 [27.6]	0.0 [0.0]	30.4 [33.8]	**83.2 [32.4]**	27.0 [34.7]
		B	95.6 [25.8]	55.6 [3.0]	63.3 [32.5]	9.5 [2.8]	56.9 [21.7]	**89.8 [7.4]**	69.4 [8.9]	75.9 [34.9]
dCPB2 3-(3,5-dichlorophenyl) benzoic acid	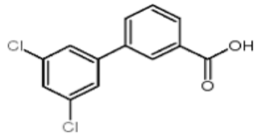 *267.1*	H	19.1 [21.2]	27.4 [28.7]	**58.2 [21.4]**	45.8 [28.8]	**51.2 [23.4]**	**46.6 [20.1]**	**54.7 [10.5]**	**56.4 [37.8]**
		P	0.0 [0.0]	3.2 [7.8]	12.2 [21.3]	0.0 [0.0]	0.0 [0.0]	0.0 [0.0]	10.1 [19.4]	2.0 [4.8]
		B	**3.6 [1.2]**	44.5 [4.0]	**30.6 [10.8]**	**6.0 [2.3]**	48.7 [19.8]	74.0 [8.6]	61.1 [4.4]	**36.1 [14.7]**
dCPB3 4-(3,5-dichlorophenyl) benzoic acid	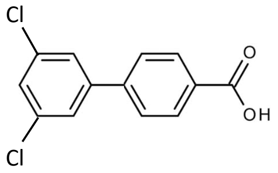 *267.1*	H	50.2 [25.7]	44.4 [25.5]	96.1 [24.9]	57.2 [32.5]	79.6 [37.7]	71.4 [47.0]	83.5 [18.7]	82.7 [40.9]
		P	0.0 [0.0]	0.0 [0.0]	8.9 [14.1]	6.0 [14.6]	0.0 [0.0]	0.0 [0.0]	0.0 [0.0]	0.0 [0.0]
		B	55.8 [20.4]	49.2 [4.0]	16.4 [6.2]	7.4 [2.4]	57.7 [21.0]	**82.0 [7.5]**	48.5 [7.3]	39.4 [15.2]
dCPB4 3-(2,5-dichlorophenyl) benzoic acid	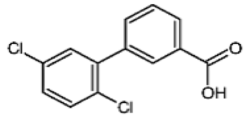 *267.1*	H	29.2 [22.7]	35.8 [24.3]	**69.1 [10.4]**	59.5 [40.0]	63.7 [28.5]	**48.8 [29.2]**	**56.5 [6.5]**	**51.3 [18.5]**
		P	0.0 [0.0]	20.5 [45.7]	8.1 [18.2]	0.0 [0.0]	0.0 [0.0]	0.7 [1.5]	6.5 [14.6]	0.0 [0.0]
		B	37.9 [6.8]	50.1 [1.9]	19.1 [9.0]	9.0 [3.2]	82.6 [40.3]	76.1 [7.7]	69.2 [4.5]	41.4 [11.9]
dCPB5 4-(2,3-dichlorophenyl) benzoic acid	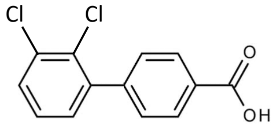 *267.1*	H	52.6 [46.6]	77.4 [7.9]	82.9 [20.7]	132.7 [39.4]	70.3 [32.7]	77.8 [22.2]	85.0 [28.7]	99.6 [21.4]
		P	0.0 [0.0]	100.1 [56.0]	**86.2 [10.7]**	**92.8 [24.0]**	0.0 [0.0]	0.0 [0.0]	20.4 [11.6]	17.1 [9.9]
		B	**416.4 [52.5]**	**124.4 [18.7]**	**62.4 [17.3]**	**88.0 [15.1]**	97.1 [26.0]	74.7 [21.2]	61.6 [15.3]	**107.1 [22.7]**
dCPB6 4-(2,5-dichlorophenyl) benzoic acid	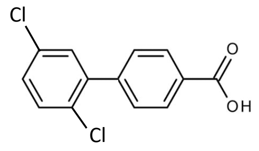 *267.1*	H	**26.1 [37.8]**	41.7 [59.0]	79.1 [35.3]	158.4 [55.7]	75.8 [34.8]	75.5 [35.6]	79.7 [28.2]	75.2 [26.3]
		P	0.0 [0.0]	24.3 [23.4]	**66.8 [18.9]**	52.8 [32.1]	0.0 [0.0]	12.2 [12.0]	13.9 [15.1]	2.7 [4.6]
		B	**335.9 [60.7]**	123.9 [36.1]	55.2 [21.9]	**78.5 [18.0]**	103.2 [25.7]	69.1 [15.6]	64.7 [21.8]	**98.5 [15.5]**

Supplemental Table S4. Hemolysis (H), proteolysis (P) and biofilm (B) for DIF and structural analogues are represented as percent of control. Mean values and standard deviation (SD) for DIF and each analogues are shown for DIF and each structural analogue. Values of structural analogues that significantly ($P < 0.05$; student's t-test) differed from DIF are indicated in bold font.

Chan et al. Supplemental Table S4D – DIF and structural analogues differentially mitigate virulence in *S. aureus*

Compound Name	Structure & M.W.		% Hemolysis (H) / Proteolysis (P) vs. Control (St. Dev.)							
			MSSA	COL	LAC	MW2	Mu3	Mu50	C15	C16
dCPB7<br>3-(2,4-dichlorophenyl) benzoic acid	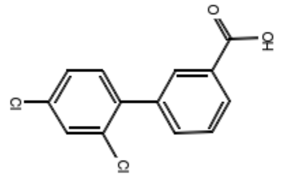<br>*267.1*	H	**24.5 [35.3]**	68.0 [13.3]	68.2 [9.6]	167.8 [71.9]	72.2 [24.2]	67.1 [29.8]	71.8 [11.8]	62.4 [35.6]
		P	0.0 [0.0]	**49.6 [5.4]**	**39.3 [13.3]**	**55.4 [17.2]**	0.0 [0.0]	0.0 [0.0]	22.9 [20.0]	22.5 [19.8]
		B	99.8 [13.6]	**120.7 [10.6]**	56.8 [19.0]	**62.8 [21.6]**	66.7 [12.7]	54.6 [12.2]	49.2 [13.0]	67.7 [15.6]
BZA<br>Benzoic Acid	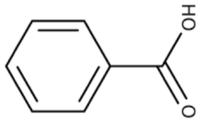<br>*122.1*	H	**147.3 [26.2]**	127.9 [21.0]	100.0 [14.3]	**168.5 [53.4]**	105.1 [18.4]	90.6 [16.4]	**102.3 [20.1]**	114.4 [7.6]
		P	127.6 [0.0]	**112.6 [34.0]**	**107.0 [15.5]**	**125.2 [19.5]**	105.4 [66.2]	**109.7 [28.4]**	88.0 [43.7]	109.0 [60.9]
		B	**129.8 [12.9]**	**107.8 [16.6]**	**89.9 [35.2]**	**103.8 [17.9]**	95.8 [23.3]	**135.0 [36.2]**	109.1 [35.1]	143.3 [55.5]
dCB1<br>2,4-Dichlorobenzoic acid	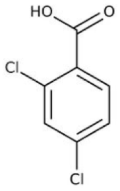<br>*191.0*	H	**146.1 [18.4]**	115.0 [20.2]	106.1 [10.5]	159.6 [40.2]	105.2 [48.0]	81.3 [11..8]	96.9 [13.0]	105.5 [30.7]
		P	44.6 [0.0]	94.6 [38.1]	**96.0 [22.3]**	**100.8 [26.8]**	51.5 [49.1]	**113.7 [43.5]**	86.3 [40.8]	81.2 [45.5]
		B	143.3 [33.6]	**124.4 [15.0]**	116.4 [62.8]	**114.8 [14.8]**	94.9 [17.8]	**119.4 [17.7]**	90.9 [23.3]	**110.9 [26.9]**
dCB2<br>3,4-Dichlorobenzoic acid	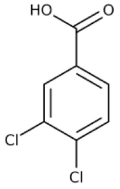<br>*191.0*	H	143.0 [36.6]	115.2 [0.2]	102.2 [24.4]	**164.9 [34.8]**	90.5 [62.1]	88.0 [23.1]	**94.2 [17.6]**	98.8 [22.2]
		P	82.9 [0.0]	**100.5 [13.8]**	**105.2 [17.2]**	**150.7 [54.6]**	16.2 [16.6]	**95.2 [29.2]**	79.0 [36.9]	104.9 [59.5]
		B	**110.2 [20.9]**	**83.4 [12.9]**	163.1 [85.8]	**104.1 [35.4]**	103.8 [48.7]	117.7 [52.8]	76.7 [25.0]	**127.2 [37.6]**
dCB3<br>2,5-Dichlorobenzoic acid	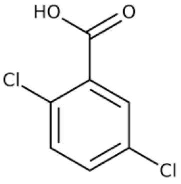<br>*191.0*	H	**139.7 [25.7]**	90.7 [34.0]	93.8 [23.1]	**128.6 [15.3]**	89.8 [36.7]	86.5 [30.4]	**105.3 [13.2]**	98.0 [32.4]
		P	19.1 [0.0]	**136.2 [54.8]**	**89.4 [10.9]**	**115.8 [22.2]**	95.8 [62.5]	**135.5 [22.9]**	103.8 [49.3]	118.1 [91.9]
		B	116.7 [12.5]	**131.2 [14.0]**	**164.0 [50.6]**	**122.4 [26.9]**	100.5 [13.3]	118.4 [33.3]	101.2 [23.0]	**104.6 [20.2]**

Supplemental Table S4. Hemolysis (H), proteolysis (P) and biofilm (B) for DIF and structural analogues are represented as percent of control. Mean values and standard deviation (SD) for DIF and each analogues are shown for DIF and each structural analogue. Values of structural analogues that significantly ($P < 0.05$; student's t-test) differed from DIF are indicated in bold font.

Chan et al. Supplemental Table S4E – DIF and structural analogues differentially mitigate virulence in *S. aureus*

Compound Name	*Structure & M.W.*		% Hemolysis (H) / Proteolysis (P) vs. Control (St. Dev.)							
			MSSA	COL	LAC	MW2	Mu3	Mu50	C15	C16
dFB1 2,6-Difluorobenzoic acid	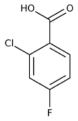 [illegible]	H	**179.9 [32.8]**	153.2 [55.5]	103.1 [18.2]	203.1 [62.8]	101.6 [57.1]	94.6 [23.2]	106.2 [25.6]	113.3 [24.4]
		P	68.6 [0.0]	117.2 [50.4]	**103.6 [13.2]**	**106.4 [13.9]**	106.6 [78.3]	99.7 [43.9]	81.1 [54.2]	99.3 [73.2]
		B	142.9 [26.6]	**156.3 [41.5]**	**231.0 [75.2]**	**133.4 [13.8]**	93.9 [9.7]	125.5 [36.3]	86.9 [31.7]	**107.3 [16.8]**
dFB2 2,3-Difluorobenzoic acid	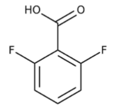 *158.1*	H	**157.7 [24.5]**	114.3 [3.3]	109.8 [13.7]	159.7 [26.2]	103.7 [22.9]	83.3 [14.2]	109.7 [27.2]	129.8 [23.2]
		P	110.9 [0.0]	**118.7 [38.7]**	**105.1 [7.3]**	**111.1 [20.2]**	92.6 [75.8]	**114.0 [8.7]**	83.6 [34.9]	107.9 [55.1]
		B	129.1 [19.3]	**108.6 [18.4]**	234.4 [110]	**135.1 [28.2]**	110.1 [36.]	**137.4 [14.7]**	101.9 [30.9]	121.3 [34.4]
dFB3 2,4-Difluorobenzoic acid	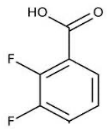 *158.1*	H	**150.4 [2.0]**	120.5 [11.1]	110.0 [11.1]	**174.1 [35.9]**	116.8 [37.1]	87.0 [18.3]	**101.2 [17.9]**	110.1 [17.2]
		P	128.2 [0.0]	**140.8 [44.5]**	**114.6 [12.4]**	**131.1 [16.6]**	103.8 [65.1]	**109.3 [10.4]**	**91.0 [21.5]**	**108.4 [38.5]**
		B	87.9 [14.1]	**143.8 [25.2]**	**81.1 [34.6]**	**106.9 [10.5]**	118.8 [37.0]	125.1 [29.6]	92.2 [20.5]	**125.6 [6.3]**
CFB 2-Chloro-4-fluorobenzoic acid	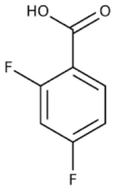 *174.6*	H	**153.7 [32.2]**	113.9 [6.7]	109.4 [14.1]	176.5 [33.7]	118.5 [33.3]	103.1 [20.9]	115.5 [29.5]	122.8 [25.5]
		P	114.8 [0.0]	**109.3 [20.9]**	**100.2 [20.8]**	**120.0 [25.7]**	104.5 [82.0]	**130.6 [19.8]**	**92.8 [35.1]**	110.4 [54.7]
		B	136.2 [28.0]	**220.7 [54.0]**	210.0 [80.4]	**131.5 [16.8]**	125.6 [36.0]	133.2 [39.4]	113.8 [33.7]	**147.7 [21.3]**

Supplemental Table S4. Hemolysis (H), proteolysis (P) and biofilm (B) for DIF and structural analogues are represented as percent of control. Mean values and standard deviation (SD) for DIF and each analogues are shown for DIF and each structural analogue. Values of structural analogues that significantly (P < 0.05; student's t-test) differed from DIF are indicated in bold font.
